# A Randomized Controlled Dose-Escalation Study of LY06006, a Recombinant Humanized Monoclonal Antibody to RANKL, in Chinese Healthy Adults

**DOI:** 10.3389/fphar.2022.893166

**Published:** 2022-06-15

**Authors:** Suping Niu, Min Chen, Diqin Yan, Xiangxing Liu, Shuren Guo, Lun Ou, Huaying Fan, Jie Lv, Qian Wang, Wenliang Dong, Lin Xia, Simin Wang, Gang Liu, Qun Gu, Danjie Guo, Hongxia Liu, Huiying Rao, Qingshan Zheng, Xiaoyan Nie, Haifeng Song, Yi Fang

**Affiliations:** ^1^ Department of Science and Research, Peking University People’s Hospital, Beijing, China; ^2^ Department of Pharmacy, Peking University People’s Hospital, Beijing, China; ^3^ Department of Pharmacy Administration and Clinical Pharmacy, School of Pharmaceutical, Peking University, Beijing, China; ^4^ School of Pharmacy, Xuzhou Medical University, Xuzhou, China; ^5^ Shandong Boan Biotechnology Co., Ltd., Yantai, China; ^6^ Beijing United-Power Pharma Tech Co., Ltd., Beijing, China; ^7^ Department of Intensive Care Units, Peking University People’s Hospital, Beijing, China; ^8^ Shanghai University of Traditional Chinese Medicine, Shanghai, China; ^9^ Peking University People’s Hospital, Peking University Hepatology Institute, Beijing Key Laboratory of Hepatitis C and Immunotherapy for Liver Disease, Beijing, China; ^10^ The Center for Drug Clinical Research of Shanghai University of TCM, Shanghai, China; ^11^ State Key Laboratory of Proteomics, Beijing Proteome Research Center, National Center for Protein Sciences (Beijing), Beijing Institute of Lifeomics, Beijing, China

**Keywords:** pharmacokinetics, pharmacodynamics, immunogenicity, denosumab, RANK/RANKL, osteoporosis

## Abstract

**Background:** This study was conducted to explore the safety, tolerance, pharmacokinetics, pharmacodynamics, and immunogenicity of LY06006, a recombinant humanized monoclonal antibody to RANKL, when administrated subcutaneously in Chinese healthy adults.

**Research design and methods:** This was a randomized, double-blinded, placebo-controlled, single ascending dose study performed in 32 healthy Chinese adults, who were randomly assigned to receive a single injection dose of 18, 60, 120 mg study drug or placebo with a follow-up of 140–252 days.

**Results:** No deaths or drug-related serious adverse events occurred. LY06006 was rapidly absorbed in the 60 mg group with a T_max_ range of 120–480 h and serum LY06006 concentrations decreased slowly 11–13 days after dosing with a long mean (SD) half-life of 389.58 (63.44) h. The most frequent AEs were elevated serum parathyroid hormone (PTH) level (83.3%), hypocalcemia (54.2%), and hypophosphatemia (45.8%). None of the 32 subjects tested positive for anti-drug antibody during the trial.

**Conclusion:** Single-dose subcutaneous administration of LY06006 was safe and well-tolerated in healthy Chinese adults. C_max_ showed linear pharmacokinetic characteristics in the dose range of 18–120 mg based on dose-exposure proportionality analysis.

## Introduction

Osteoporosis, defined as reduced bone mineral density (BMD), deteriorated bone microarchitecture, and reduced bone strength, is a major contributor to hip fractures and vertebral fractures which are often referred to as osteoporosis fractures (Compston et al., 2019). Osteoporosis fractures are one of the most common causes of disability (Cummings and Melton, 2002). Notably, women after menopause are at increased risk of osteoporosis fracture. Approximately 50% of women will have at least one fracture after the age of 50 years (Jones et al., 1994).

Receptor activator kappa-B ligand (RANKL) is essential for the formation, function, and survival of osteoclasts (Boyle et al., 2003). By binding to its receptor RANK on the surface of osteoclasts and osteoclast precursors, it can result in an increase in bone resorption through the final differentiation and activation of osteoclasts.

Denosumab (Prolia®) is a fully human monoclonal antibody (IgG_2_) against RANKL preventing the combination of RANKL and RANK(Cummings et al., 2009). It has been proved that denosumab reduced bone resorption and increased BMD, which are associated with a significant reduction in the risk of osteoporosis fractures in postmenopausal women (Cummings et al., 2009; Papapoulos et al., 2012; Bone et al., 2013; Nakamura et al., 2014; Bone et al., 2017).

LY06006, a recombinant humanized monoclonal antibody against RANKL, was a potential biosimilar to denosumab. A high degree of similarity between LY06006 and Prolia® has been observed in bioactivity, pharmacokinetics, pharmacodynamics and pharmacotoxicity in preclinical studies (data not published).

This phase 1 study was conducted to explore the safety, tolerance, pharmacokinetics, pharmacodynamics and immunogenicity of LY06006 when administrated subcutaneously in healthy adults.

## Subjects and Methods

### Study Design

This was a randomized, double-blinded, placebo-controlled, single ascending dose study in 32 healthy Chinese adults at phase I department, Peking University People’s Hospital (China). The study was conducted in compliance with the Declaration of Helsinki, Guideline for Good Clinical Practice, and was approved by the Ethics Committee of Peking University People’s Hospital (No. 2017PHA-090-01). Written informed consent was obtained from each subject before the initiation of the investigation. The study was registered at the Chinese Clinical Trials Registry (registration number: CTR 20171459).

Pharmaceutical and pre-clinical studies showed that the no observed adverse effect level (NOAEL) of LY06006 in the toxicological study of multiple administration in cynomolgus monkeys is 10 mg/kg. In the light of the guidelines issued by FDA, the calculated initial dose should not exceed 19.2 mg based on the mean weight of 60 kg of healthy adults (FDA, 2005). Therefore, we set the initial dose as 18 mg. According to the clinical recommendation dosage and previous clinical studies of Prolia^®^ in healthy adults, three groups were set with the dose of 18 mg, 60 mg, and 120 mg, respectively (Mcclung et al., 2006; Bekker et al., 2004; Kumagai et al., 2011; Chen et al., 2018).

Subjects were randomly assigned (3:1) to LY06006 or placebo in each group. Only after tolerance evaluation on day 56 of the lower dose group was completed and safety and tolerance were confirmed, the next dose group could be carried out. All subjects were not allowed to be given calcium and Vitamin D supplements throughout the study period. Such preparations could be used as appropriate only after the occurrence of serum calcium reduction (<2.0 mmol/L) with relevant clinical symptoms.

Screening occurred 30 days before dosing. Eligible subjects received a single abdominal subcutaneous injection of the investigational product or placebo on day 0 (D0) and a sequent 4-days hospitalization for observation. Subjects were discharged on day 4. After that, a follow-up period continued during which subjects returned to the hospital for safety evaluations and blood sampling for pharmacokinetics (PK), pharmacodynamics (PD), and immunogenicity assessments. Subjects in 18, 60, and 120 mg groups were followed for 4 months (D140), 6 months (D196), and 9 months (D252), respectively.

### Subjects

Healthy adults aged 18–65 years with a body mass index of 19.0–24.0 kg/m^2^ were eligible. Exclusion criteria contain: 1) suffered or ongoing osteomyelitis, jaw necrosis, odontopathy, or maxillary disease in an active stage needing oral surgery; 2) administration of any medications that might affect bone turnover (e.g., denosumab, bisphosphonates or fluoride within 12 months, estrogens, selective estrogen receptor modulators, parathyroid hormone, systemic glucocorticosteroids, Vitamin D supplements (>1000 IU/day), anabolic steroids, calcitriol or available analogs, or diuretics within 6 months, and inhaled or topical glucocorticoid within 2 weeks); 3)recent bone fracture (within 6 months); 4) had acute or chronic infections; 5) hypocalcemia, hypercalcemia, or abnormal serum albumin-adjusted blood calcium levels; 6) history of drug abuse, smoking or alcohol; 7) pregnant or breastfeeding women.

### Safety Assessments

Safety assessment was based on the monitoring of adverse events (AEs) and serious AEs (SAEs) along with routine clinical and laboratory assessment, including physical examination, vital signs, blood tests, urinalysis, and 12-lead electrocardiograms. Safety evaluations were conducted at baseline and regularly throughout the study.

The frequency, severity, and characterization of AEs were continuously recorded using Medical Dictionary for Regulatory Activities (MedDRA version 22.0). AE intensity was graded according to the National Cancer Institute Common Terminology Criteria for Adverse Events (CTCAE) version 4.03.

### Pharmacokinetics Assessments

Blood samples for PK evaluation were collected at 1 h preceding the injection, and at 6 h (D1), 24 h (D2), 72 h (D4), 120 h (D6), 168 h (D8), 216 h (D10), 264 h (D12), 312 h (D14), 480 h (D21), 648 h (D28), 984 h (D42), 1320 h (D56), 1656 h (D70), 1992 h (D84), 2328 h (D98), 2664 h (D112), 3336 h (D140) after dosing in 18 mg groups. Blood samples collected at 4008 h (D168), 4680 h (D196) were added in 60 mg group and 4008 h (D168), 4680 h (D196), 5352 h (D224), 6024 h (D252) were added in 120 mg group.

15 min of centrifugation was needed to separate the supernatant serum, and these serum samples were stored at −80°C before analysis. Serum levels of LY06006 were detected by Electrochemiluminescence assay (ECLA). The lower limit of quantification (LLOQ) was 39.06 ng/ml and the quantitative range was 39.06–5000 ng/ml. Values determined lower than the LLOQ were set to zero.

The pharmacokinetic parameters, the area under the plasma drug concentration-time curve (AUC), the time taken to reach the maximum concentration (T_max_), peak concentrations of drug in serum (C_max_), terminal elimination half-life (t_1/2z_), apparent volume of distribution (V_d_/F) and the apparent clearance (CL_z_/F) were calculated using the plasma concentration-time data collected. The pharmacokinetic properties of LY06006 were assessed based on drug concentrations and basic pharmacokinetic parameters calculated.

### Immunogenicity Assessments

Blood samples were collected at pre-dose (within 1 h) and days 14, 28, 42, 56, 84, 140, 196 (only 60 and 120 mg groups), and 252 (only 120 mg groups) after the end of injection to determine anti-drug antibodies (ADAs) and neutralizing antibodies (NAbs).

Validated bridging ECLA was applied for ADA measurement, and negative control response criteria (NRC) was 70.7 electrochemiluminescence unit. The antibody response criteria (ARC) of low concentration positive control and high concentration positive control were 1.59 and 150.80, respectively. The signal-to-noise ratio (S/N) of the screening cut-point was 1.11, and the confirmatory cut point (CCP) of confirmation results was 10.9%.

### Pharmacodynamics Assessments

The plasma concentrations of C-terminal telopeptide of type I collagen (CTX-1), bone-specific alkaline phosphatase (BALP), and parathyroid hormone (PTH) were reported as pharmacodynamic parameters. Pharmacodynamic blood collection points were the same as the pharmacokinetic blood collection time of the corresponding group.

CTX-1, BALP, and PTH were determined by enzyme-linked immunosorbent assays (ELISA). Plasma levels of PTH were detected by ECLA. The detection method was verified by recording its specificity, quantitative limit (LOQ), goodness-of-fit/linearity, accuracy, and precision (intra-assay and inter-assay). The linear range of CTX-1 and BALP are 0.117–2.00 and 7.00–90.0 ng/ml, respectively. All values less than the LLOQ were reported as the value of LLOQ.

### Statistical Analysis

Statistical significance was set at *p* < 0.05 for all tests. Non-compartmental pharmacokinetics analysis for LY06006 was conducted using Phoenix® WinNonlin® 7.0 (Certara, Princeton, NJ, USA) and other statistical analyses were analyzed using SAS (Statistical Analysis System; SAS Institute Inc, Cary, NC, United States, version 9.4).

The full analysis set (FAS) included all subjects who were randomly allocated. The safety set (SS) included all subjects who received randomization and study drug, with safety evaluation data. The PK concentration set (PKCS) included all subjects who received the study drug and had ≥1 quantifiable plasma concentration collected after dosing. The PK parameter set (PKPS) included all subjects who received the study drug dose and had ≥1 valid PK parameter. FAS was used for the analysis of the demographic characteristics. SS was used for safety analysis. PKCS was used for the plasma concentration versus time statistics analysis. And PKPS were used for PK parameters analysis.

The plasma concentration versus time statistics was depicted by semi-log concentration-time curves. The mean, standard deviation, coefficient of variation, quartile, maximum, minimum, and geometric mean of PK parameters of each group were calculated. Differences across treatment groups in PK parameters including C_max_, AUC, t_1/2z_, V_d_/F, and CL_z_/F were analyzed using analysis of variance (one-way ANOVA), while T_max_ was examined by Kruskal–Wallis H test. Meanwhile, all PK parameters except T_max_ were log-transformed in statistical analysis. A multiple comparisons test with Bonferroni correction was performed when necessary. Dose-exposure proportionality was assessed using a power model. The assumption was that the logarithm of the PK variable (C_max_ and AUC) is linearly related to the logarithm of dose: ln (PK) = β_0_ + β_1_ *ln (dose). Dose proportionality was established when the 90% CI for the slope β_1_ fell completely within the range 0.882–1.118 (the criterion interval: 1 + [ln (0.80)/ln(r)], 1 + [ln (1.25)/ln(r)], r = the highest dose/the lowest dose) (Smith et al., 2000).

## Results

### Demographic Characteristics of the Participants

**FIGURE 1:**
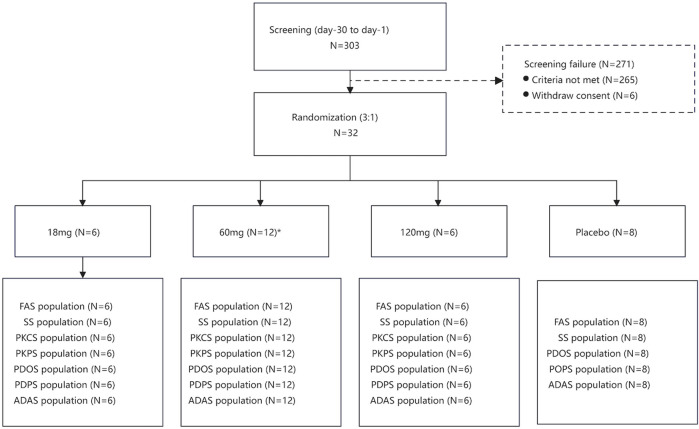
Flow chart of the study.

Between 9 January 2018, and 13 May 2019, of 303 individuals assessed for eligibility, 32 were allocated into three groups (Figure 1). 31 participants completed the trial but one participant received 60 mg of study drug did not complete the trial because of pregnancy. Data from the participant who did not complete the study were included in the FAS, SS, PKCS, PKPS, PDOS, PDPS and ADAS. A summary of baseline characteristics is provided in Table 1. Baseline characteristics were comparable between each group.

**TABLE 1:** Demographic characteristics of the participants.

	Groups, dose (mg)			
Characteristic	18 (*N* = 6)	60 (*N* = 12)	120 (*N* = 6)	Placebo (*N* = 8)
Age, years				
Mean (SD)	30.7 (8.82)	26.4 (4.62)	36.0 (8.29)	28.8 (6.67)
Range	22–43	19–37	24–44	23–44
Gender, n (%)				
Male	4 (66.7)	8 (66.7)	2 (33.3)	5 (62.5)
Female	2 (33.3)	4 (33.3)	4 (66.7)	3 (37.5)
Ethnicity, n (%)				
Han	6 (100.0)	11 (91.7)	6 (100.0)	8 (100.0)
Others	0	1 (8.3)	0	0
Occupation, n (%)				
Physical	0	3 (25.0)	1 (16.7)	1 (12.5)
Non-physical	6 (100.0)	9 (75.0)	5 (83.3)	7 (87.5)
Weight, kg				
Mean (SD)	62.08 (6.722)	62.25 (5.715)	59.65 (6.817)	55.79 (5.896)
BMI, kg/m^2^				
Mean (SD)	22.80 (1.083)	21.62 (1.647)	22.30 (0.860)	21.24 (1.328)

SD, standard deviation; BMI, body mass index. The body-mass index is the weight in kilograms divided by the square of the height in meters.

### Safety Results

No deaths or drug-related serious adverse events (SAEs) occurred. No adverse events (AEs) led to withdrawal from the trial, except for 1 pregnancy. Subsequent post hoc sensitivity analyses showed that the overall incidence of AEs was not affected before and after removing the pregnancy event.

259 treatment-emergent adverse events (TEAEs) were reported in 24 participants receiving LY06006 and 29 TEAEs were reported in seven participants who received a placebo. All were mild or moderate in intensity and resolved. The overall incidence and classification of TEAEs are summarized in Table 2.

**TABLE 2:** Summary of adverse events (excluding pregnancy event).

Dose (mg)	18 (*N* = 6)	60 (*N* = 12)	120 (*N* = 6)	Study drug (*N* = 24)	Placebo (*N* = 8)
	*n* (%)	*n* (%)	*n* (%)	*n* (%)	*n* (%)
TEAEs	6 (100)	12 (100)	6 (100)	24 (100)	7 (87.5)
Grade1	2 (33.3)	2 (16.7)	2 (33.3)	6 (25.0)	4 (50.0)
Grade2	2 (33.3)	7 (58.3)	3 (50.0)	12 (50.0)	3 (37.5)
Grade3	1 (16.7)	1 (8.3)	1 (16.7)	3 (12.5)	0
Grade4	1 (16.7)	2 (16.7)	0	3 (12.5)	0
Grade5	0	0	0	0	0
SAEs	0	0	0	0	0

The most frequent AEs were elevated serum PTH level (83.3%), hypocalcemia (54.2%), and hypophosphatemia (45.8%). AEs that occurred frequently are listed in Table 3.

**TABLE 3:** Common treatment-emergent adverse events.

	Dose group (mg)				
Teae, n (%)	18 (N = 6)	60 (N = 12)	120 (N = 6)	Study drug (N = 24)	Placebo (N = 8)
Total	6 (100.0)	12 (100.0)	6 (100.0)	24 (100.0)	7 (87.5)
Investigations					
PTH level elevated	5 (83.3)	10 (83.3)	5 (83.3)	20 (83.3)	0
TBil level elevated	1 (16.7)	3 (25.0)	2 (33.3)	6 (25.0)	3 (37.5)
CK level elevated	2 (33.3)	3 (25.0)	1 (16.7)	6 (25.0)	2 (25.0)
AST level elevated	2 (33.3)	3 (25.0)	1 (16.7)	6 (25.0)	1 (12.5)
ALT level elevated	2 (33.3)	3 (25.0)	0	5 (20.8)	1 (12.5)
DBil level elevated	0	2 (16.7)	2 (33.3)	4 (16.7)	2 (25.0)
White blood cell count decreased	0	3 (25.0)	2 (33.3)	5 (20.8)	0
Triglyceride level elevated	0	4 (33.3)	0	4 (16.7)	1 (12.5)
Glucose level elevated	1 (16.7)	2 (16.7)	1 (16.7)	4 (16.7)	1 (12.5)
Neutrophil count decreased	0	3 (25.0)	2 (33.3)	5 (20.8)	0
Uric acid elevated	0	3 (25.0)	0	3 (12.5)	1 (12.5)
White blood cell count increased	0	2 (16.7)	0 (0)	2 (8.3)	1 (12.5)
Red blood cell sedimentation rate increased	1 (16.7)	0	1 (16.7)	2 (8.3)	1 (12.5)
Lymphocyte count decreased	0	1 (8.3)	1 (16.7)	2 (8.3)	1 (12.5)
GGT level elevated	0	1 (8.3)	0	1 (4.2)	1 (12.5)
Neutrophil count increased	0	1 (8.3)	0	1 (4.2)	1 (12.5)
Metabolism and nutrition disorders					
Hypocalcemia	4 (66.7)	6 (50.0)	3 (50.0)	13 (54.2)	2 (25.0)
Hypophosphatemia	3 (50.0)	6 (50.0)	2 (33.3)	11 (45.8)	0
Hypercholesterolemia	1 (16.7)	0	0	1 (4.2)	2 (25.0)
Infections and infestations					
Upper respiratory tract infection	1 (16.7)	4 (33.3)	1 (16.7)	6 (25.0)	1 (12.5)
Pericoronitis	0	3 (25.0)	1 (16.7)	4 (16.7)	0
Periodontitis	0	0	1 (16.7)	1 (4.2)	1 (12.5)
Gastrointestinal disorders					
Oral ulcer	0	2 (16.7)	1 (16.7)	3 (12.5)	0
General disorders and administration site conditions					
Injection site swelling	3 (50.0)	0	0	3 (12.5)	0
Injection site erythema	3 (50.0)	0	0	3 (12.5)	0
Blood and lymphatic system disorders					
Anemia	0	0	3 (50.0)	3 (12.5)	0

Listed are treatment-emergent adverse events that had been reported in at least 10% of the participants. PTH: parathyroid hormone; TBil: total bilirubin; CK: creatine kinase; AST: aspartate aminotransferase; ALT: alanine aminotransferase; DBil: direct bilirubin; GGT: Gamma-glutamyl transferase.

### Pharmacokinetic Results

**FIGURE 2:**
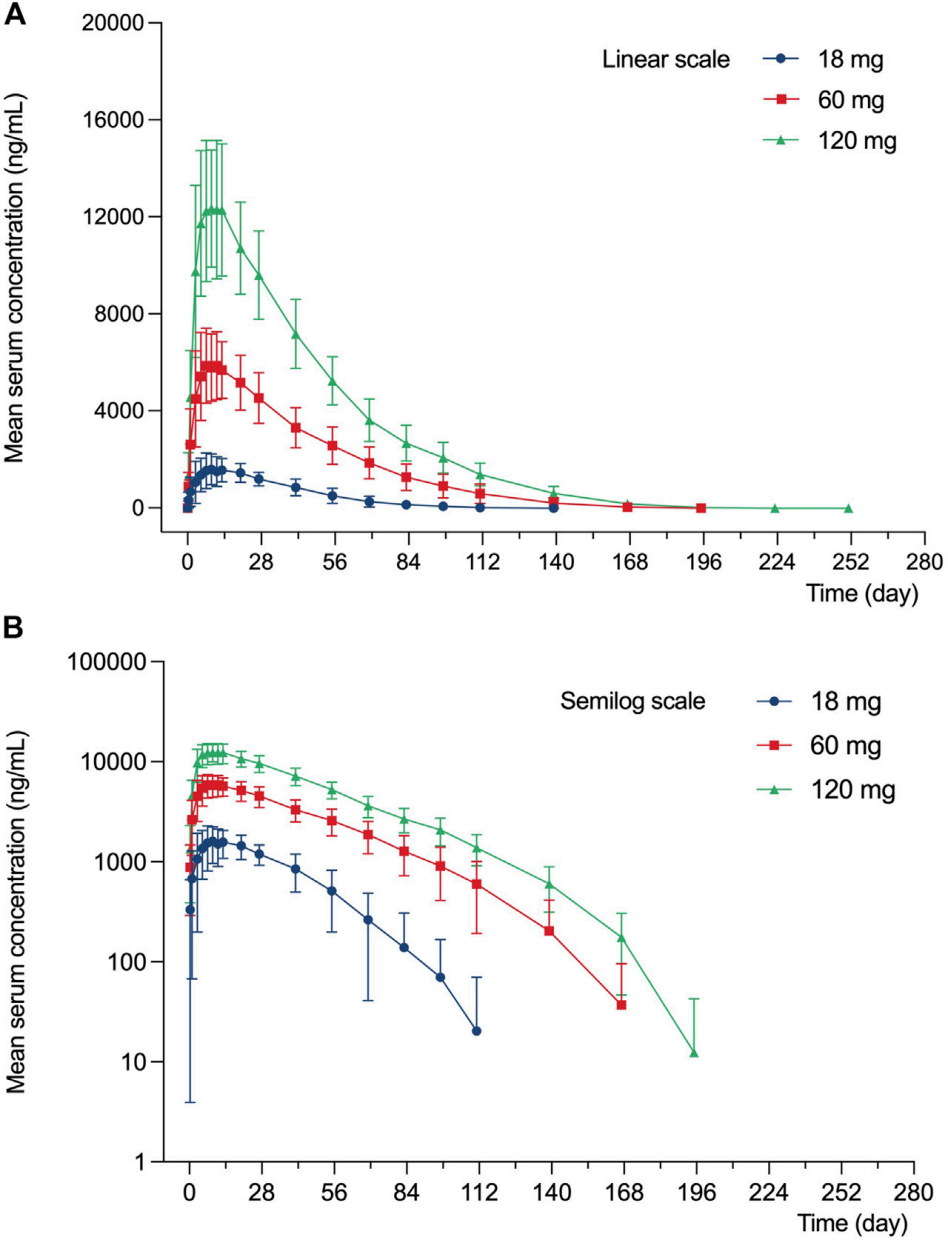
Mean (SD) serum concentration profile of LY060006 **(A)** Linear scale. **(B)** Semilogarithmic scale.

The serum concentration-time profile of LY06006 is displayed in Figure 2.

LY06006 was rapidly absorbed with detectable concentrations observed 6 h after the dose in all groups. The T_max_ was relatively long, with a range of 120–480 h for all dose groups and serum LY06006 concentrations decreased slowly 11–13 days after dosing with a long half-life ranging from 168 to 216 h. Concentration profiles over time showed a biphasic decline that a slow initial phase characterized by an approximately linear decline in serum concentration followed by a more rapid elimination phase.

Pharmacokinetics parameters from the noncompartmental analysis of LY06006 (Table 4) showed that with the dose increased, the exposure to LY06006 increased, which was reflected by both AUC_0–t_ and C_max_. In addition, the median t_1/2z_ of LY06006 was also observed prolonged with the escalation of dose. The mean CL_z_/F decreased from 11.42 to 7.41 from 18 to 120 mg. The mean t_1/2z_ rose from 343.91 to 470.84 from 18 to 60 mg. MRT_0-t_ was 692.87 h (18 mg), 945.38 h (60 mg) and 1009.91 h (120 mg).

**TABLE 4:** Pharmacokinetics parameters of single injection of LY06006.

PK parameters	18 mg	60 mg	120 mg
	(*N* = 6)	(*N* = 12)	*(N* = 6)
t_1/2z_ (h)	343.91 (128.30)	389.58 (63.44)	470.84 (84.08)
C_max_ (ng/ml)	1735.00 (599.82)	6,370.83 (1449.29)	12,986.67 (2,652.75)
T_max_(h)	216.00 (120–480)	192.00 (120–480)	168.00 (120–264)
AUC_0-t_ (day*ng/mL)	70,254.66 (25,914.14)	324,149.75 (92,030.23)	694,679.00 (147,765.48)
AUC_0-∞_(day*ng/mL)	72,305.88 (26,811.83)	328,125.73 (91,973.46)	699,802.36 (147,559.97)
AUC_0-day140_ (day*ng/mL)	72,007.48 (26,483.46)	322,745.11 (88,090.96)	683,363.50 (140,528.85)
V_z_/F (ml)	5272.79 (1677.31)	4,531.35 (1276.22)	5119.67 (1731.81)
CL_z_/F (ml/h)	11.42 (3.39)	8.34 (2.88)	7.41 (1.56)
MRT_0-t_ (h)	692.87 (173.23)	945.38 (147.42)	1009.91 (82.62)
MRT_0-∞_ (h)	746.08 (194.76)	981.25 (143.17)	1037.17 (76.68)
AUC__%Extrap_ (%)	2.78 (1.46)	1.31 (0.76)	0.76 (0.44)

Values are presented as mean (SD); T_max_ is reported as the median (range). Note that the symbol “*” represents multiplication.

The dose-proportionality analysis (Table 5) found that values of β of C_max_ (90% CI: 0.947–1.207), AUC_(0-∞)_ (90% CI: 1.070–1.374), and AUC_0-day140_ (90% CI: 1.063–1.361) were partially within the criterion interval 0.882–1.118.

**TABLE 5:** C_max_, AUC_0-Day140_, AUC_0-∞_, and dose relationship for single injection of 18–120 mg LY06006 (based on 90%CI).

Parameters	Estimate	SE	90%CI	Statistics	*p* Values
C_max_ (ng/ml)					
β_0_	4.311	0.304	3.789–4.833	14.176	<0.001
β_1_	1.077	0.076	0.947–1.207	14.257	<0.001
AUC_0-∞_ (day*ng/mL)					
β_0_	7.626	0.355	7.016–8.236	21.452	<0.001
β_1_	1.222	0.088	1.070–1.374	13.837	<0.001
AUC_0-day140_ (day*ng/mL)					
β_0_	7.653	0.349	7.054–8.251	21.950	<0.001
β_1_	1.212	0.087	1.063–1.361	13.991	<0.001

0.80–1.25 Linear range after dose transformation: 0.882–1.118. Note that the symbol “*” represents multiplication.

### Immunogenicity Results

All participants (32, 100%) had negative ADA test results within 1 h before administration. No participants tested positive for anti-drug antibodies after a single subcutaneous injection of LY06006 injection/placebo, and during follow-up.

### Pharmacodynamic Results

**FIGURE 3:**
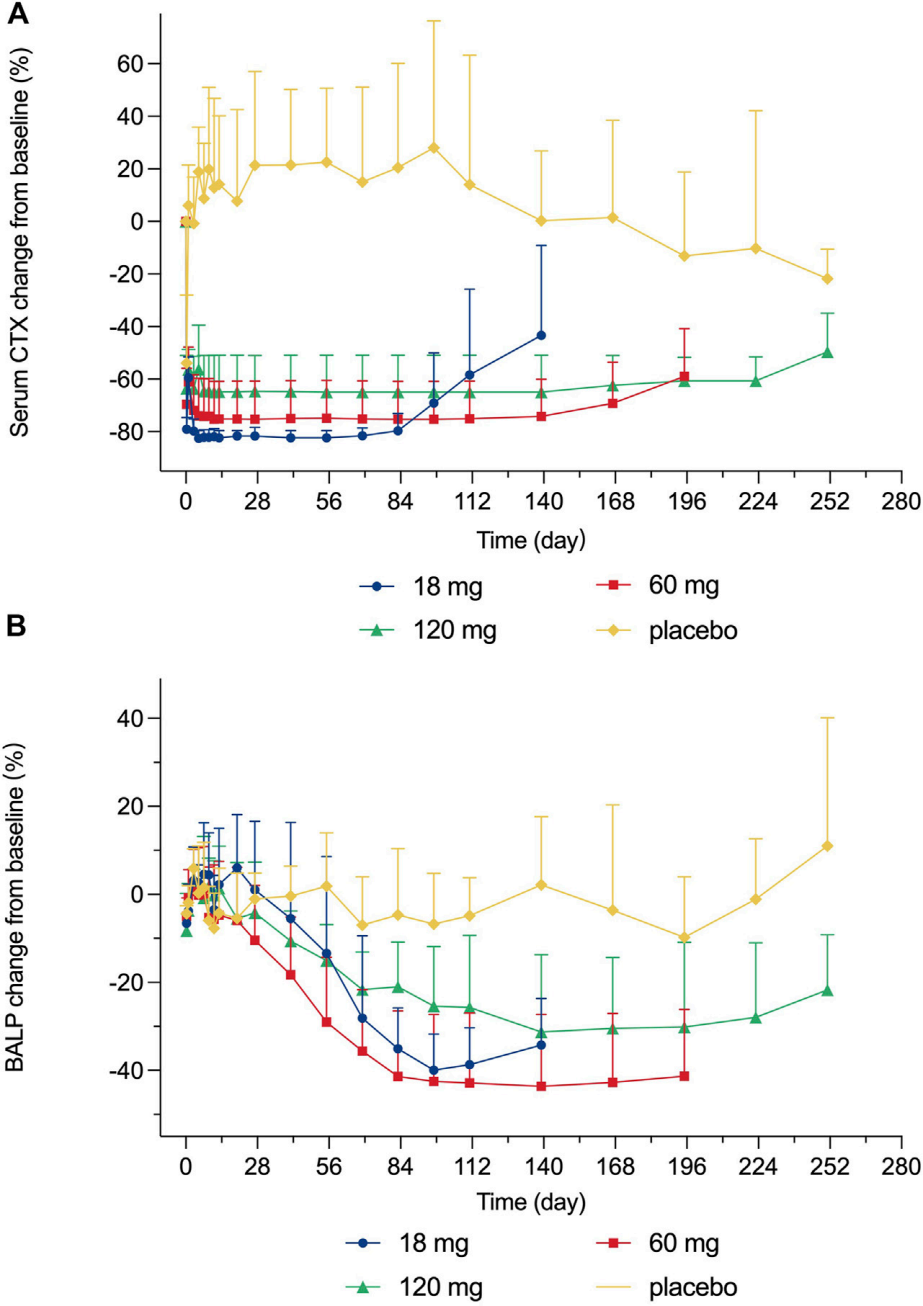
Percent change from baseline in serum **(A)** CTX-1 concentration **(B)** BALP concentration.

After a single subcutaneous injection of 18–120 mg LY06006 injection in healthy subjects, the serum CTX-1 concentration showed a consistent trend over time (Figure 3). A rapid decrease of serum CTX-1 was observed, which was able to be detected as early as 6 h after administration with a decline of 79.05% (4.50), 69.62% (13.70), and 63.55% (12.57) from baseline in 18 mg, 60 mg, and 120 mg group, respectively, while the decline in the placebo group was 53.91% (25.88). The CTX-1 decrease was positively correlated with the dose, based on 18 mg LY06006 could maintain the inhibition level to 84 days, 60 mg to 140 days, 120 mg to 224 days, and then slowly increased. Besides, the serum CTX-1 concentration did not recover to the baseline level during the whole observation period. The mean decline (SD) of the 18 mg (D140), 60 mg (D196) and 120 mg (D252) group was 43.37% (34.25), 58.94% (18.19) and 49.60% (14.69).

Serum BALP concentration showed a consistent trend over time after a single subcutaneous injection of 18–120 mg LY06006 in healthy subjects (Figure 3). The mean (SD) maximum BALP inhibition of the 18 mg group was 39.93% (8.21) on day 98 after administration, and was maintained until day 140. The mean (SD) inhibition of BALP in the 60 mg group reached 41.34% (14.85) on day 84 after administration, and the mean (SD) maximum inhibition reached 43.63% (16.36) on day 140 after administration and remained until day 196. BALP inhibition of the 120 mg dose group reached the maximum inhibition level of 31.30% (17.62) on day 140 after administration and could maintain until day 252. BALP concentration did not return to the baseline level during the observation period. The mean (SD) of the 18 mg group was 34.25% (10.57) on day 140 and the 60 mg group was 41.33% (15.15) on day 196. The 120 mg group had a mean (SD) reduction of 21.72% (12.53) from baseline on day 252.

**FIGURE 4:**
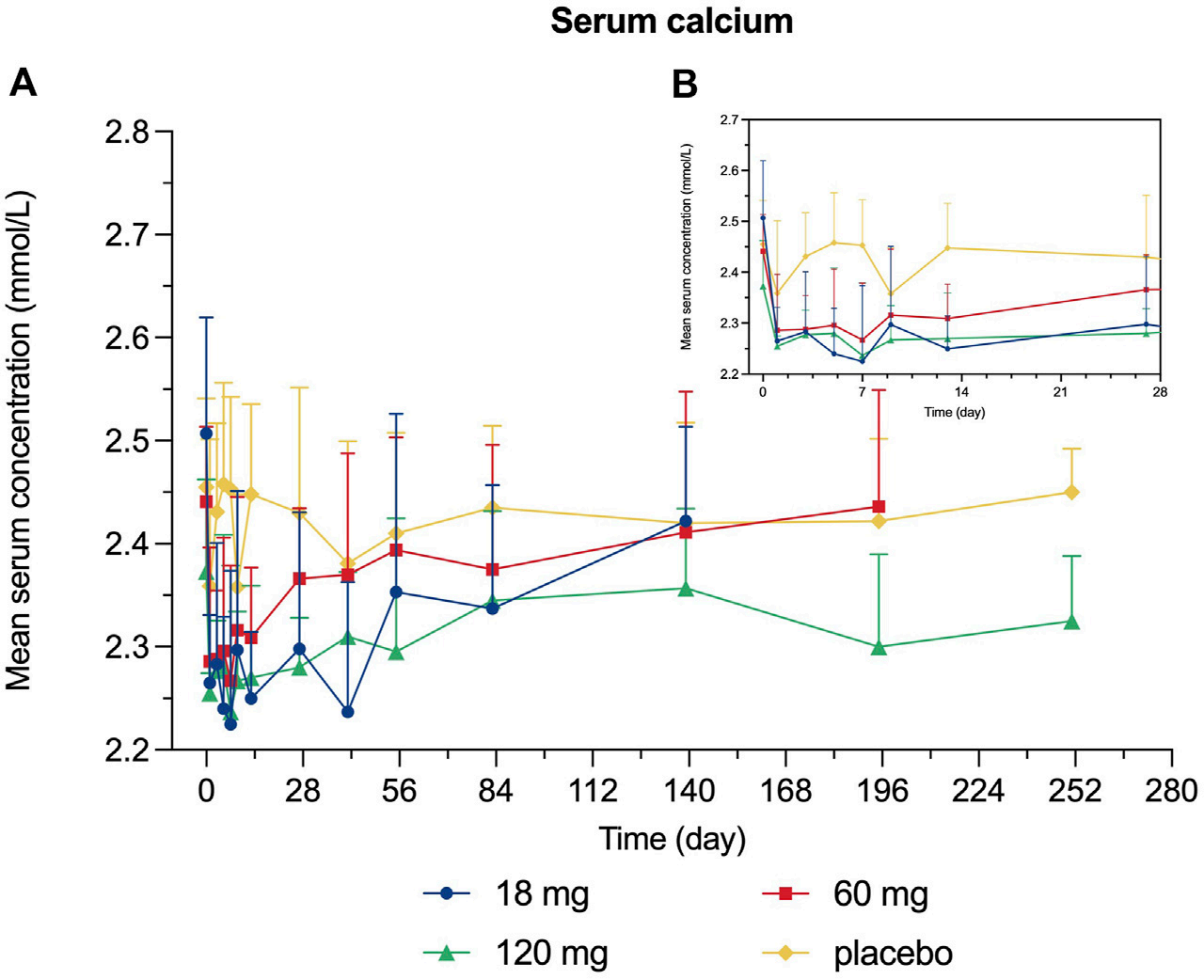
Percent change from baseline in serum calcium. **(A)** day 0∼day 252; **(B)** day 0∼day 28.

**FIGURE 5:**
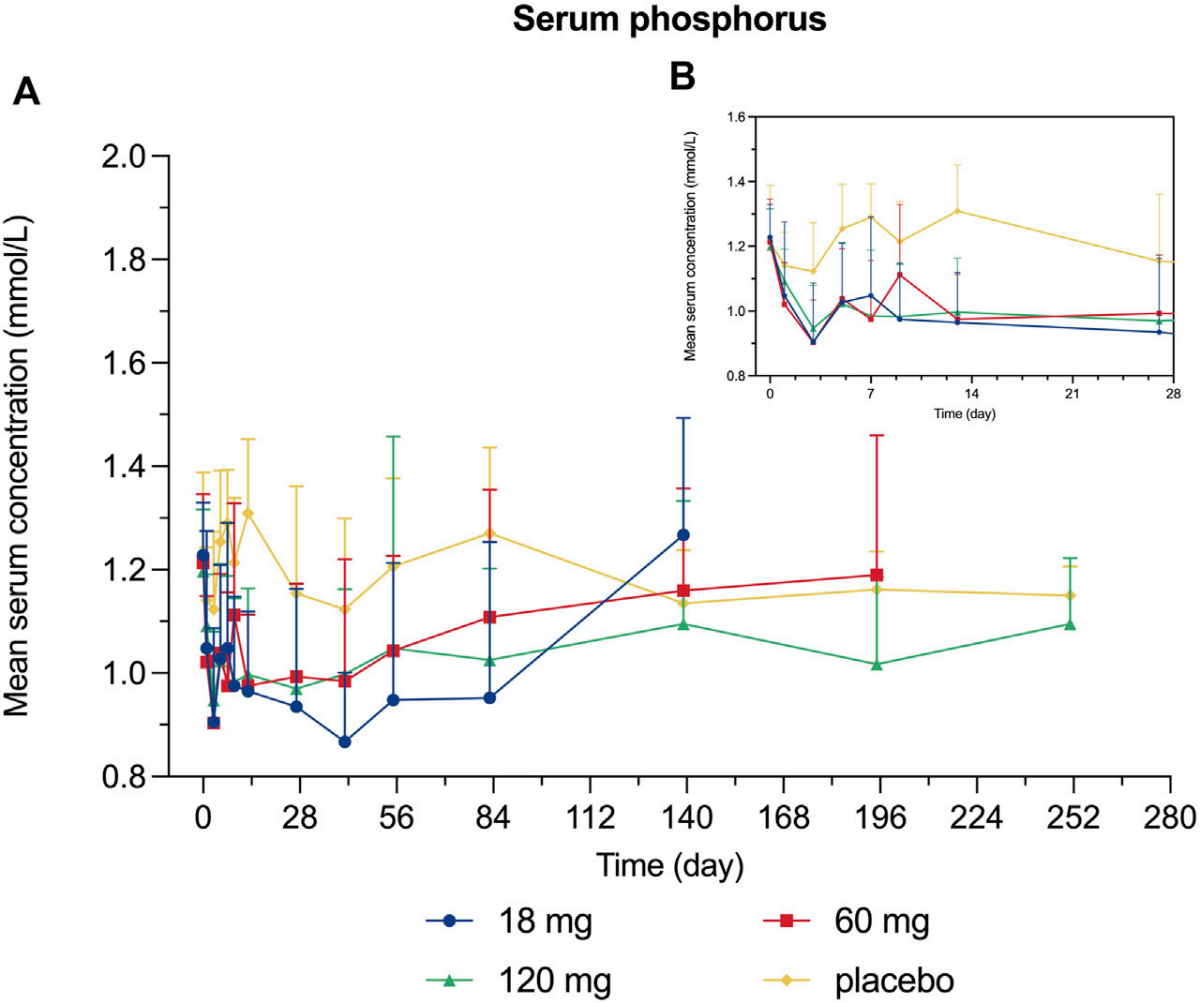
Percent change from baseline in serum phosphorus. **(A)** day 0∼day 252; **(B)** day 0∼day 28.

**FIGURE 6:**
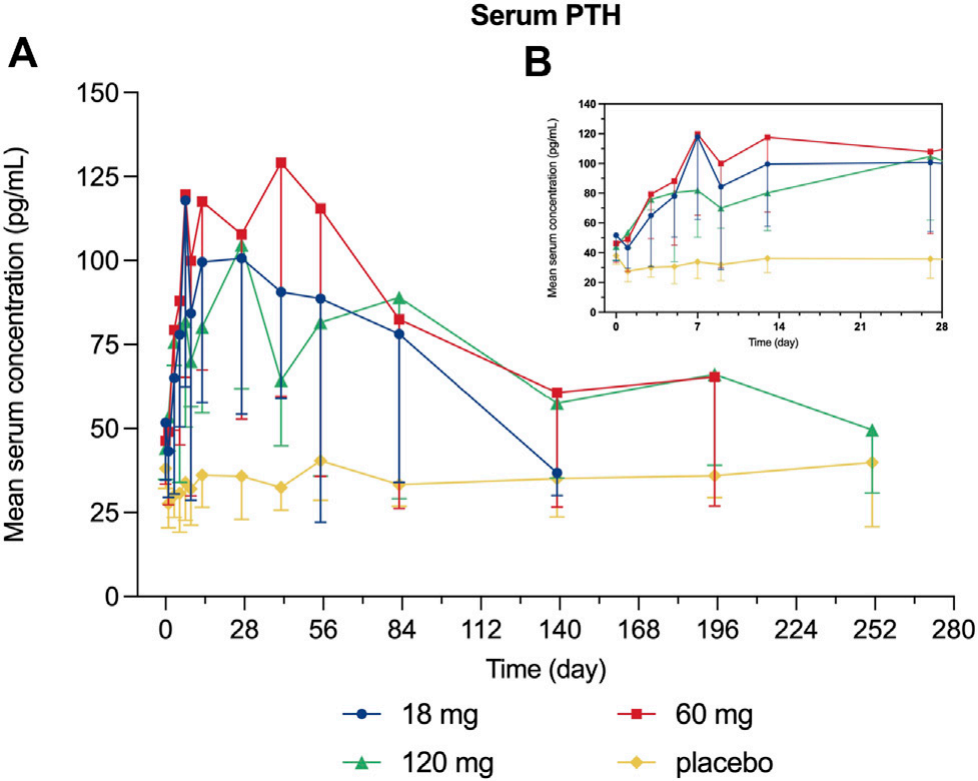
Percent change from baseline in serum PTH. **(A)** day 0∼day 252; **(B)** day 0∼day 28.

Decreased levels of serum calcium and phosphorus were observed shortly after injection, with the maximum mean decrease observed on day 7 and day 3, respectively (Figures 4, 5). Serum PTH increased with the maximum mean increase observed on day 7 (Figure 6).

## Discussion

This study provided the first-in-human data sets of LY06006. In this study, the safety, tolerability, PK, and PD of single subcutaneous administration of LY06006 were assessed in healthy Chinese participants. These results proved LY06006 to be well-tolerated, and no significant safety issues have been identified in this study.

There was no serious adverse event happened and the severity of most AEs is slight (grade 1–2). The most common AEs observed, as shown in Table 3, were elevated PTH levels (83.3%), hypocalcemia (54.2%), and hypophosphatemia (45.8%), which were consistent with previous studies of denosumab and were also listed in the label of Prolia®. Such AEs had been reported in the clinical trials of KN012, a biosimilar of Prolia®, with the incidence of low calcium level and low phosphate level being 80.8 and 61.5%, respectively (Zhang et al., 2021). Another study also showed similar results that a decline in blood calcium level occurred in 72.8% of healthy adults who were given a biosimilar of denosumab (Zhang H. et al., 2020). It is noteworthy that participants in our study were required not to take calcium or vitamin D supplements during the study unless serum calcium was reduced to less than 2.0 mmol/L and accompanied by clinical signs and symptoms, which could be used after a comprehensive evaluation by the investigator. In addition, none of the participants received calcium or vitamin D supplements throughout the trial. Such instruction was given not only to ensure that hypocalcemia events would not be covered up but also to enable healthy subjects to provide the most sensitive assessment of PK since previous studies showed a potential decrease in serum calcium levels associated with denosumab (Bekker et al., 2004; Kumagai et al., 2011; Chen et al., 2018).

Since bone remodeling is a dynamic process consisting of osteoclast-mediated bone resorption and osteoblast-mediated bone formation, this mechanism allows bones to act as an adequate reservoir of calcium and phosphorus (Zhang N. et al., 2020). Hence the decreased calcium level could probably be explained that denosumab inhibits bone resorption by binding to RANKL, thus inhibiting the mobilization of stored calcium and phosphate, leading to less bone calcium and phosphorus release, eventually resulting in hypocalcemia and hypophosphatemia (Dempster et al., 2012). We measured the serum calcium, serum phosphorus, and serum PTH of participants and demonstrated their mean value curves over time. Transient decreases in serum calcium and phosphorus occurred right after LY06006 was administered and slowly return to the baseline level, which is consistent with its antiresorptive effect (Figures 4, 5). The serum PTH increased a little later in response to the decrease in serum calcium concentration and reached its peak concentration 7 days after injection, which could be considered a functional regulation of PTH and is crucial for calcium homeostasis (Figure 6) (Kraenzlin and Meier, 2011). Therefore, hypocalcemia may be exacerbated by the use of LY06006 and pre-existing hypocalcemia must be corrected prior to initiating therapy with LY06006. Clinical monitoring of calcium and mineral levels (e.g., phosphorus) is highly recommended within 14 days of LY06006 injection. Besides, calcium and vitamin D supplementation should be given when necessary to maintain calcium levels, as listed on the label of Prolia®(Amgen, 2021).

The PK properties of denosumab (Prolia®) in postmenopausal women at different doses (0.01–3 mg/kg) have been clearly described in previous studies (Bekker et al., 2004). A long elimination and a more rapid terminal elimination were identified with serum denosumab concentrations being observed decreasing at a faster rate when the serum denosumab concentration was less than approximately 1 μg/ml (Bekker et al., 2004). This nonlinear PK properties of denosumab could be well described by a target-mediated drug disposition model in which target-mediated elimination pathway starts to become saturated with the increase of serum denosumab concentration, leading to the decrease of total clearance (Marathe et al., 2008; An, 2020). In the studies of subsequent biosimilars of denosumab, similar PK characteristics were observed (Zhang H. et al., 2020; Zhang et al., 2021; Chen et al., 2022). In our study, the t_1/2_ value increased from 14 to 19 days with the drug dose increased from 18 to 60 mg and the exposure (AUC and C_max_) of 120 mg LY06006 was more than twofold of 60 mg LY06006. The concentration-time curve (Figure 2) dropped more sharply when serum concentration was below 100 ng/ml, which was in accordance with the nonlinear PK of denosumab. The PK parameters in our work, C_max_, AUC_0-∞_, and AUC_0-day140_ were dose-proportioned by the power function model. The 90% CI of β_1_ values of C_max_, AUC_0-∞_, and AUC_0-day140_ were 0.947–1.207, 1.070–1.374, and 1.063–1.361, respectively (Table 5). The 90% CI of the β_1_ values did not completely fall in the range after dose transformation (0.882–1.118). However, due to the crossover between the 90% CI of β_1_ value and the range, it cannot be concluded whether LY06006 possesses linear or nonlinear pharmacokinetic characteristics in the dose range of 18–120 mg.

In order to exploratory and analyze the effect of LY0600 on the early clinical stage, bone turnover markers were used as PD indicators instead of fractures and bone mineral density on the basis of existing guidelines (Cosman et al.,(Cosman et al., 2014), Kanis et al., 2019, Qaseem et al., 2017). The greater the short-term decrease in bone turnover marker levels, the greater the long-term increase in bone mineral density (Eastell and Szulc, 2017). Besides, lower bone turnover marker level was related to less fracture risk (Vasikaran et al., 2011).

The PD indicators in our study consisted of CTX-1 (bone resorption marker) and BALP (bone formation marker). There was a rapid and dramatic decrease in CTX-1 which is believed to reflect the activity of osteoclasts (Figure 3). BALP levels remained closed to the baseline for about 28 days until showed an obvious decline. Although the duration of inhibition in CTX-1 and BALP was dose-dependent, the magnitude of inhibition did not show dose-dependent. For example, the maximum mean (SD) inhibition of CTX-1 was 82.48 (2.95)% in the 18 mg group, 75.25 (14.41)% in the 60 mg group, and 64.90 (14.04) % in the 120 mg group. One possible explanation is that the detected CTX-1 levels of many subjects during the follow-up period were less than the LLOQ (0.117 ng/ml) and thus reported as the value of LLOQ. This statistical method made the CTX-1 change from baseline correlated with its baseline level in the different dose groups, while the latter was highest in the 18 mg group, second in the 60 mg group and lowest in the 120 mg group. The maximum mean (SD) inhibition of BALP was 39.93 (8.21)% in the 18 mg group, 43.63 (16.36)% in the 60 mg group, and 31.30 (17.62)% in the 120 mg group. The magnitude of changes in serum calcium, serum phosphorus, and serum PTH change did not reflect dose-dependent, and the magnitude difference between dose groups was not significant either.

Combined with data from pre-clinical studies that there was no dose-correlation between serum β-CTX, serum osteocalcin (OST), and serum procollagen I N-terminal peptide (P1NP) at 1, 3, and 10 mg/kg of LY06006 in 3-months repeated toxicity tests, these data may indicate that LY06006 has bound with all the hRANKL after injection. In other words, LY06006 may have reached target saturation at the dose of 18 mg. Therefore, the PD indicators did not reflect a dose-related magnitude of change, since higher doses may not be related to a more significant effect.

The immunogenicity was analyzed. The overall ADA-positive rates were 0% (0/32) before and after injection, which was consistent with that denosumab demonstrated low immunogenicity in humans (Amgen, 2021).

## Conclusion

1Single-dose subcutaneous administration of LY06006 was safe and well-tolerated in healthy Chinese adults. C_max_ showed linear pharmacokinetic trend in the dose range of 18–120 mg but dose-exposure of LY06006 could not be concluded as nonlinear. Serum CTX-1 concentration showed a consistent trend over time, and the maintenance time of CTX-1 inhibition was positively correlated with the dose. The serum BALP concentration exhibited a slow decreasing trend. None of the subjects tested positive for ADA during the trial.

## Data Availability

The raw data supporting the conclusion of this article will be made available by the authors, without undue reservation.
